# Balancing functions of annexin A6 maintain equilibrium between hypertrophy and apoptosis in cardiomyocytes

**DOI:** 10.1038/cddis.2015.231

**Published:** 2015-09-03

**Authors:** P Banerjee, V Chander, A Bandyopadhyay

**Affiliations:** 1 Cell Biology and Physiology Division, Council of Scientific and Industrial Research – Indian Institute of Chemical Biology, 4, Raja SC Mullick Road, Kolkata 700 032, West Bengal, India

## Abstract

Pathological cardiac hypertrophy is a major risk factor associated with heart failure, a state concomitant with increased cell death. However, the mechanism governing progression of hypertrophy to apoptosis at the single-cell level remains elusive. Here, we demonstrate annexin A6 (Anxa6), a calcium (Ca^2+^)-dependent phospholipid-binding protein critically regulates the transition of chronic hypertrophied cardiomyocytes to apoptosis. Treatment of the H9c2(2-1) cardiomyocytes with hypertrophic agonists upregulates and relocalizes Anxa6 with increased cytosolic punctate appearance. Live cell imaging revealed that chronic exposure to hypertrophic agonists such as phenylephrine (PE) compromises the mitochondrial membrane potential (ΔΨ_m_) and morphological dynamics. Such chronic hypertrophic induction also activated the caspases 9 and 3 and induced cleavage of the poly-(ADP-ribose) polymerase 1 (Parp1), which are the typical downstream events in the mitochondrial pathways of apoptosis. An increased rate of apoptosis was evident in the hypertrophied cardiomyocytes after 48–72 h of treatment with the hypertrophic agonists. Anxa6 was progressively associated with the mitochondrial fraction under chronic hypertrophic stimulation, and Anxa6 knockdown severely abrogated mitochondrial network and dynamics. Ectopically expressed Anxa6 protected the mitochondrial morphology and dynamics under PE treatment, and also increased the cellular susceptibility to apoptosis. Biochemical analysis showed that Anxa6 interacts with Parp1 and its 89 kDa cleaved product in a Ca^2+^-dependent manner through the N-terminal residues (1–28). Furthermore, expression of Anxa6^S13E^, a mutant dominant negative with respect to Parp1 binding, served as an enhancer of mitochondrial dynamics, even under chronic PE treatment. Chemical inhibition of Parp1 activity released the cellular vulnerability to apoptosis in Anxa6-expressing stable cell lines, thereby shifting the equilibrium away from cell death. Taken together, the present study depicts a dual regulatory function of Anxa6 that is crucial for balancing hypertrophy with apoptosis in cardiomyocytes.

Complex machineries govern the life and death decisions in mammalian cells through a dynamic equilibrium, which is essential for physiological homeostasis.^1^ Such equilibrium is critical for cardiac myocytes because of their terminally differentiated states and low proliferative capacities. Stress response in cardiomyocytes often involves a switch between survival and cell death pathways.^2, 3, 4^ Cardiomyocyte hypertrophy is an adaptive response to stress, which may turn maladaptive and fatal,^5^ as evident in cardiovascular disorders that leads to heart failure.^6^ Hypertrophied phenotypes are also associated with a balance between cell growth and programmed cell death.^7^ These processes are aided by several patrolling proteins, which sense and operate to ameliorate the anomalies.^8, 9^ Understanding the dynamics of such signaling events is vital for the development of novel therapeutic strategies.

Anxa6 belongs to the annexin family of calcium (Ca^2+^)/phospholipid-binding proteins.^10^ A major cardiac annexin,^11^ Anxa6 has diverse functions ranging from handling intracellular Ca^2+^ signaling, cholesterol transport,^12^ Ras inactivation^13^ and vesicular traffic.^14^ Anxa6 mostly functions as an intracellular scaffold.^15^ Although mice with targeted depletion of the *Anxa6* gene remain viable,^16^ functional redundancies within the annexin family have been proposed to compensate for the loss of Anxa6 function.^17, 18^ A 10-fold overexpression of Anxa6 targeted to the heart developed cardiomyopathies in mice, whereas cardiomyocytes from Anxa6-knockout mice exhibited increased contractility and altered Ca^2+^ turnover.^19, 20^ Such contradictory findings may indicate participation of Anxa6 in counterbalancing signaling mechanisms. Moreover, end-stage heart failures have been reported to be associated with downregulation of Anxa6, and, in general, Anxa6 has compensatory roles in chronic pathological conditions.^20, 21, 22^ However, the function of differential Anxa6 expression or dynamics in chronic cardiomyocyte hypertrophy is poorly understood.

We have reported the interactions of Anxa6 with the sarcomeric *α*-actinin and its role in cardiomyocyte contractility.^23^ Recently, we have characterized a role of Anxa6 in the antihypertrophic signaling via the regulation of atrial natriuretic peptide (ANP) secretion.^24^ The mechanistic spectrum of Anxa6 in the earlier study was limited to a short-term (24 h) exposure of H9c2 cardiomyocytes to the *α*1-adrenergic receptor agonist phenylephrine (PE). The dynamics of Anxa6 within this small window yielded valuable insight into the spatiotemporal regulation of hypertrophic signaling. Here, we extended the study to understand the dynamics of Anxa6 under chronic hypertrophic conditions. The mechanodeficient H9c2(2-1) cardiomyocyte line has been instrumental in our study to rule out the contributions of Anxa6 towards contractility,^23^ owing to its multidimensional scaffold activity and functional compensations.^17, 18^ The H9c2 cardiomyocytes have been extensively characterized and ARE an established animal origin-free model for studying signal-transduction pathways in cardiomyocytes, including hypertrophy.^25, 26^

Adrenergic stimulation is crucial in compensatory and pathological cardiac hypertrophy, an early state that may proceed towards heart failure.^27^ Cardiac hypertrophy at advanced stages (chronic) is associated with mitochondrial dysfunction, which also contributes to cardiac decompensation.^28^ To explore the temporal events under chronic hypertrophy, we analyzed the effects of adrenergic induction on mitochondrial membrane potential (ΔΨm) and morphological dynamics, parameters that are directly correlated with mitochondrial dysfunction and programmed cell death.^29, 30, 31^ Anxa6 has been reported to be associated with mitochondria in some cell types.^17, 32, 33^ In the present study, we aim to understand the functions of Anxa6 under chronic hypertrophic conditions that may progress towards apoptosis.

## Results

### Chronic hypertrophy of H9c2 cardiomyocytes is concomitant with altered expression and localization dynamics of Anxa6

**Figure 1:**
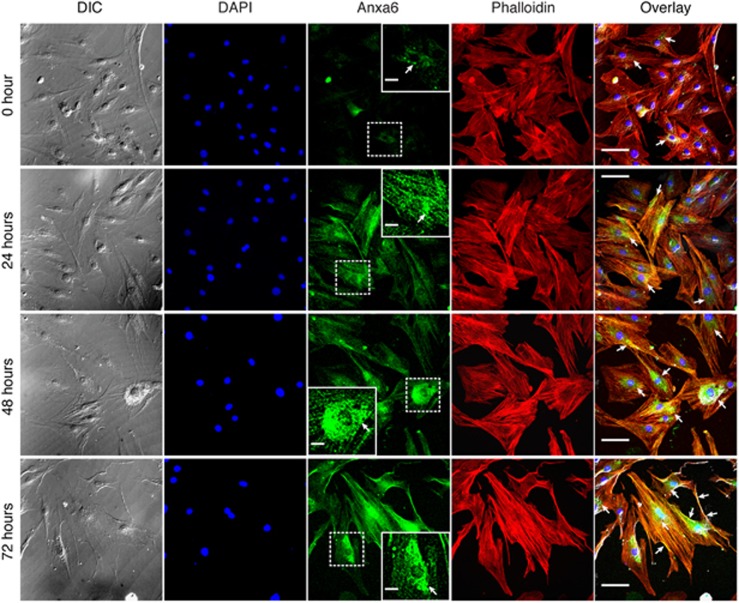
Relocalization dynamics of Anxa6 with chronic hypertrophic development. H9c2 cardiomyocytes were treated with 100 μM PE for 72 h and were costained for Anxa6 and F-actin by ICC-IF (pseudocolored green) and Alexa Fluor 546 phalloidin (pseudocolored red), respectively, at indicated time points. Nucleus was counterstained with DAPI (4',6-diamidino-2-phenylindole; pseudocolored blue). Overlay represents maximum intensity projections of RGB channels merged with DIC. Arrowheads, distribution of punctate signals. Confocal images at × 600 total magnification. Scale bar, 40 *μ*m. Insets, punctate structures in the juxtanuclear compartment. Scale bar, 5 *μ*m

**Figure 2:**
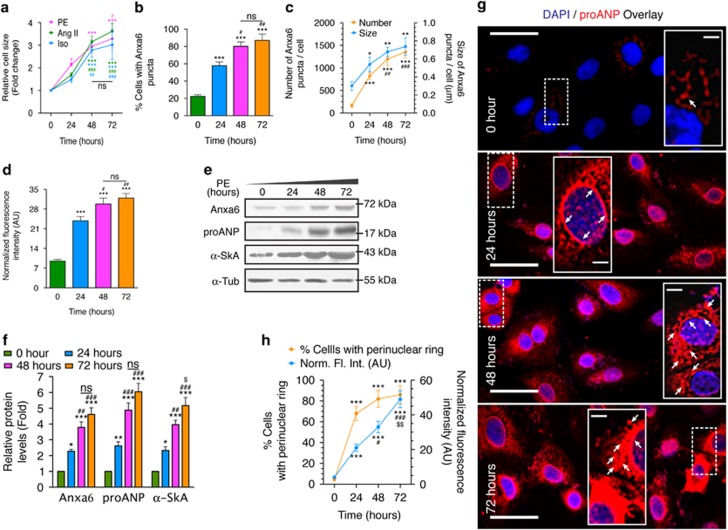
Quantitative assessment of Anxa6 dynamics in cardiomyocytes with chronic hypertrophied phenotypes. H9c2 cells treated with 100 μM PE or 200 nM Ang II or 10 μM Iso for 72 h were analyzed for hypertrophy-associated transformations. (**a**) Quantification of cell size by staining with Alexa Fluor 546 phalloidin, as shown in Figure 1; *n*=50 cells, ***, ^###^*P*<0.0001; **^, ##^*P*<0.01; *^, #^*P*<0.05 (*, *t*=0; #, *t*=24; NS=not significant). (**b**) Percentage of cells with Anxa6 puncta, in the course of treatment with PE; *n*=10 fields, ***P<0.0001; ^##^*P*<0.01; ^#^*P*<0.05 (*, *t*=0; #, *t*=24; NS=not significant). (**c**) Number (left Y axis) and diameter (right Y axis) of Anxa6 punctate structures from Figure 1; *n*=30 cells; ***^, ###^*P*<0.0001; **^, ##^*P*<0.01; *P<0.05 (*, *t*=0; #, *t*=24). (**d**) Anxa6 expression analysis measured from immunofluorescent staining of cells shown in Figure 1; *n*=30 cells; ***P<0.0001; ^##^*P*<0.01; ^#^*P*<0.05 (*, *t*=0; #, *t*=24; NS=not significant). (**e**) Immunoblot: protein levels of Anxa6, pro-ANP and α-SkA. *α*-Tubulin (Tub): loading control. (**f**) Quantification of blots shown in (**e**); *n*=3, ^***, ###^*P*<0.0001; **^, ##^*P*<0.01; *, ^$^*P*<0.05 (*, *t*=0; #, *t*=24, $, *t*=48; NS=not significant). (**g**) PE-treated cells immunostained for pro-ANP. Nuclei counterstained with DAPI (4',6-diamidino-2-phenylindole). Total magnification × 630. Scale bar, 40 *μ*m. Insets, juxtanuclear pro-ANP clusters. Arrowheads, perinuclear signal. Scale bar, 3 *μ*m. (**h**) Plot showing the percentage of cells expressing perinuclear pro-ANP ring (left Y axis) and expression level of pro-ANP measured by normalized fluorescence intensity (right Y axis) of red channel from (**g**); *n*=30 cells; ***^, ###^*P*<0.0001; ^$$^*P*<0.01; ^#^*P*<0.05 (*, *t*=0; #, *t*=24; $, *t*=48)

H9c2 cardiomyocytes treated with PE were assessed for cellular hypertrophy by actin staining at the indicated time points (Figure 1). Compared with treatment with the hypertrophic agonists angiotensin II (Ang II) or isoproterenol (Iso), PE exhibited a linear response with time (Figure 2a), and was used in subsequent experiments. Increase in cell size was significant between 24 and 48 h, which reached a plateau between 48 and 72 h (Figure 2a). Imaging of cells using atomic force microscope showed prominent increase in cellular height compared with control at 48 h (Supplementary Movies 1 and 2). Staining pattern for Anxa6 at 0 and 24 h time points confirmed our earlier results,^24^ depicting punctate cytosolic and membrane localization (Figure 1). The number of cells with Anxa6 puncta increased significantly with the duration of treatment (Figure 2b), parallel to an increase in the number and size of the punctate structures (Figures 2b and c). Immunofluorescence as well as western blot analysis revealed that increased cell size was accompanied with elevated expression of Anxa6, as well as the hypertrophy markers pro-ANP (propeptide of ANP/intracellular precursor of ANP) and *α*-SkA (*α*-skeletal muscle actin), which further confirmed augmented hypertrophic responses at 48 and 72 h (Figures 2d–f). Immunostaining for pro-ANP also revealed perinuclear accumulation of ANP secretory granules that significantly increased with the duration of the treatment (Figures 2g and h).

### Chronic hypertrophic induction abrogated mitochondrial dynamics in H9c2 cardiomyocytes

**Figure 3:**
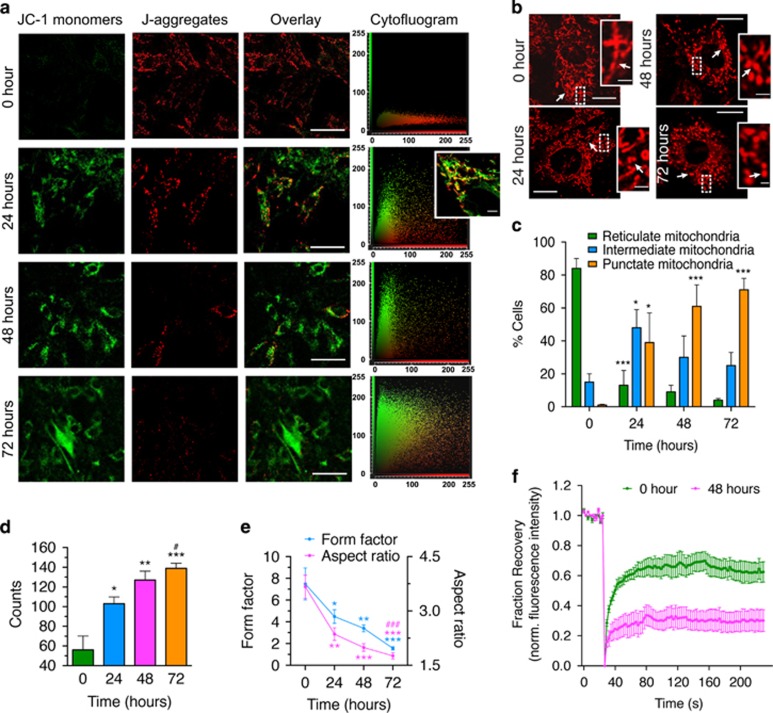
Chronic exposure to hypertrophic agonists compromises ΔΨm. H9c2 cardiomyocytes treated with 100 μM PE or 200 nM Ang II or 10 μM Iso for 72 h were analyzed for ΔΨm and dynamics in response to hypertrophic stimuli. (**a**) Confocal micrographs: JC-1 staining. Green channels display mitochondria with compromised ΔΨm and red channels show mitochondria with positive ΔΨm. Right panels: cytofluorogram. Total magnification × 630. Scale bar, 30 *μ*m. Inset: Overlay of a single cell displaying mitochondrial heterogeneity of ΔΨm. Scale bar, 5 *μ*m. (**b**) Confocal micrographs: mitochondrial morphology. Total magnification × 630. Scale bar, 10 *μ*m. Inset shows magnified view of the dotted box. Scale bar, 5 *μ*m. Arrowheads, gradual appearance of fragments from branched networks across time periods. (**c–e**) Percentage of cells with particular mitochondrial morphology (**c**); variation in the total number of mitochondria per cell (**d**); form factor (branching) and aspect ratio (length) (**e**). (**f**) FRAP assay showing relative mobility of the mitochondria in cells loaded with MitoTracker red. Plot represents normalized fluorescence intensity profiles before, during and after bleaching (recovery fraction) for *n*=16 cells per group

As the difference in cell size between 48 and 72 h was not significant (Figure 2a), we questioned whether the prohypertrophic signaling switches off during that period or culminates into other phenotypes. Hypertrophic signaling significantly overlaps with intrinsic/mitochondrial apoptotic pathways, without necessarily terminating into apoptosis.^34^ However, the transition from compensatory hypertrophy to heart failure is associated with increased cardiomyocyte death.^2, 35^ This raises the possibility of a dynamic equilibrium between hypertrophy and apoptosis, which may act as a feedback loop for cellular homeostasis. We found that chronic stimulation of H9c2 cardiomyocytes with the hypertrophic agonists PE, Ang II or Iso compromised ΔΨm, as revealed by a significant drop in tetramethylrhodamine (TMRM) fluorescence (Supplementary Figure 1A) as well as diminished red/green ratio of the potential-dependent dye JC-1, in the PE-treated cells (Figure 3a and Supplementary Figure 1B). Immunostaining of COX I (anti-OxPhos complex IV subunit I) displayed reduced tubular mitochondria, with prominent appearance of punctate structures (Supplementary Figure 1C) after 24 h of PE treatment. Live cell imaging of the chronically treated cells loaded with MitoTracker red further enabled classification of the morphology into reticulate or branched tubular networks, intermediate fragments and punctate structures with reduced mitochondrial connectivity and motility (Supplementary Figure 1D and Movie 3). Accordingly, we quantified three classes of mitochondrial morphology in chronically hypertrophied H9c2 cardiomyocytes (Figure 3b), which displayed progressive mitochondrial fragmentation with duration of PE treatment (Figures 3c–e and Supplementary Figure 1E). Furthermore, Fluorescence recovery after photobleaching (FRAP) assay on mitochondria-targeted RFP (mito-RFP) revealed delayed and diminished recovery of mitochondrial fluorescence in the agonist-treated cells, which suggested that the altered morphological dynamics of mitochondria severely abrogated its connectivity in the hypertrophied cardiomyocytes (Figure 3f).

### Chronic hypertrophic stimulation of cardiomyocytes activates intrinsic cell death signaling

**Figure 4:**
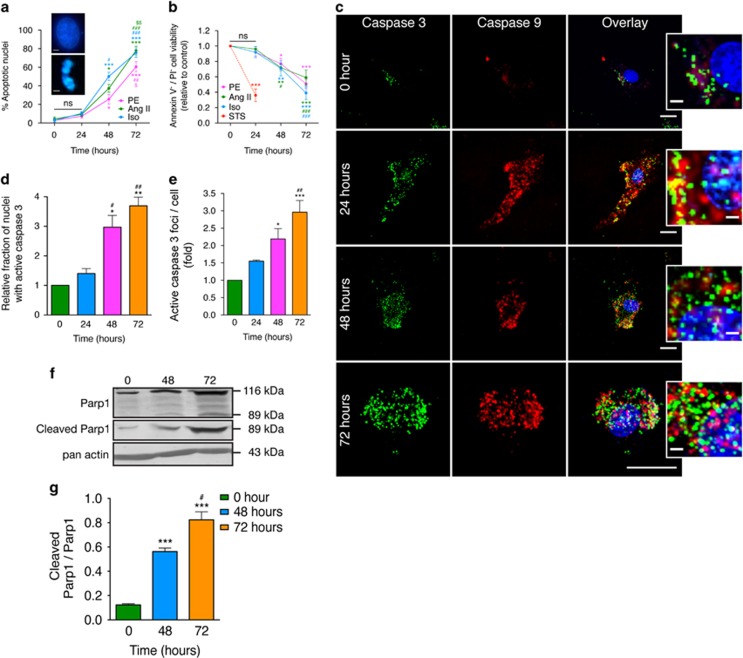
Activation of intrinsic/mitochondrial cell death pathways in response to chronic hypertrophic induction. H9c2 cardiomyocytes treated with hypertrophic agonists were assessed for activation of cell death signaling. (**a**) Percentage of cells with condensed nuclei; *n*=200 cells per group, ***^, ###^*P*<0.0001, ^##^*P*<0.01; *^, #, $^*P*<0.05 (*, *t*=0; ^#^, *t*=24; $, *t*=48, NS=not significant). Inset, normal (top) and apoptotic (bottom) nuclei. (**b**) H9c2 cell viability in response to chronic hypertrophic treatments. Cells were double stained with Alexa Fluor 488 Annexin V and PI. Staurosporin (STS) was used as a positive control; *n*=3 experiments, ***^, ###^*P*<0.0001, **P<0.01, *^, #^*P*<0.05 (*, *t*=0; ^#^, *t*=24; NS=not significant). (**c**) Confocal micrographs: distributions of active caspases 3 (green) and 9 (red), as revealed by immunostaining with antibodies against cleaved caspases 3 and 9. Total magnification × 600. Scale bar, 10 *μ*m. Insets, magnified view of nuclear/perinuclear compartment showing distribution of active caspase signal. Arrowheads, caspase-3 foci. Scale bar, 2 *μ*m. (**d** and **e**) Plots showing the fraction of nuclei with active caspase-3 (**d**) and the fraction of active caspase-3 foci per nucleus (**e**) during PE treatment; *n*=50, ^+++,^ ***P<0.0001; **, ^##^*P*<0.01; *P<0.05 (*, *t*=0; ^#^, *t*=24). (**f**) Immunoblot showing protein levels of Parp1 and cleaved Parp1 in WCL prepared at the indicated time points after PE treatment. Pan-actin was used as a loading control. (**g**) Ratiometric quantification of Parp1 activity by densitometry analysis of blots shown in (**g**); *n*=3, ***P<0.0001, ^#^*P*<0.05 (*, *t*=0; #, *t*=24)

Abrogation of ΔΨm and morphological dynamics are hallmark features of mitochondrial health and cellular susceptibility to apoptosis.^36^ However, the short-term/24 h treatments with PE, Ang II or Iso did not evoke significant apoptotic phenotypes. Apoptosis gets triggered after 48 h (Supplementary Figure 1F and Figures 4a and b), or follows after a ‘chronic' treatment period. Chronic treatments for 48 and 72 h caused significant elevation of ‘initiator' and ‘executioner' caspases 9 and 3, respectively. Active caspase-9 exhibited a more diffuse phenotype throughout the cell, whereas caspase-3 was more punctate with distinct foci in the nuclear/perinuclear compartments (Figures 4c and e). Nuclear accumulation of caspase-3 has been described as an indicator of apoptotic progression, where it is known to cause Parp1 cleavage and subsequent late-stage apoptotic effects.^37^ Immunoblot analysis from cells chronically treated with PE revealed increased Parp1 activation, denoted by increased 89 kDa cleaved Parp1 fragments (Figures 4f and g).

### Anxa6 knockdown abrogates mitochondrial dynamics in chronic cardiomyocyte hypertrophy

**Figure 5:**
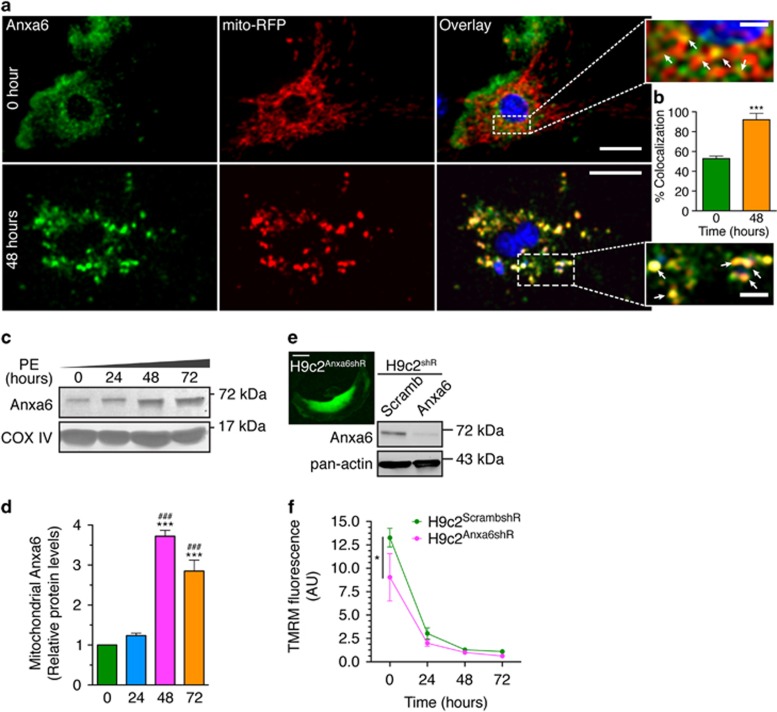
Increased mitochondrial localization of Anxa6 in cardiomyocytes protects against loss of mitochondrial dynamics under chronic hypertrophic induction. H9c2 cardiomyocytes treated with PE were analyzed for Anxa6–mitochondria association and mitochondrial dynamics. (**a**) Confocal micrographs of cells immunostained for Anxa6 (green) and mito-RFP (red). Nuclei counterstained with DAPI (4',6-diamidino-2-phenylindole; blue). Total magnification × 600. Scale bar, 10 *μ*m. Insets, colocalized clusters (yellow pixels). Scale bar, 2 *μ*m. Arrowheads, yellow overlapped pixels. (**b**) Percentage of colocalization from (**a**); *n*=25 cells; ***P<0.0001. (**c**) Immunoblot: protein levels of Anxa6 in mitochondrial fraction from cells treated with PE for indicated time periods. COX IV was used as a loading control for mitochondrial fraction. (**d**) Quantification of blot in (**c**), showing Anxa6 protein levels from mitochondrial fractions. Fold changes in PE over *t*=0; *n*=3; ***^, ###^*P*<0.0001. (**e**) Immunoblot: Anxa6 knockdown in stable H9c2 cell lines (H9c2^shR^) for Anxa6 shRNA (H9c2^Anxa6shR^), compared with scrambled (H9c2^ScrambshR^). Pan-actin was used as a loading control. Inset, a typical H9c2^Anxa6shR^ cell expressing tagGFP as a marker of transfection. Magnification × 600. Scale bar, 10 *μ*m. (**f**) TMRM-stained H9c2^ScrambshR^ (Scramb) and H9c2^Anxa6shR^ (Anxa6) cells. Magnification, × 630. Scale bar, 20 *μ*m. (**g**) TMRM fluorescence levels measured from cells shown in (**f**); *n*=50 cells, *P<0.05

**Figure 6:**
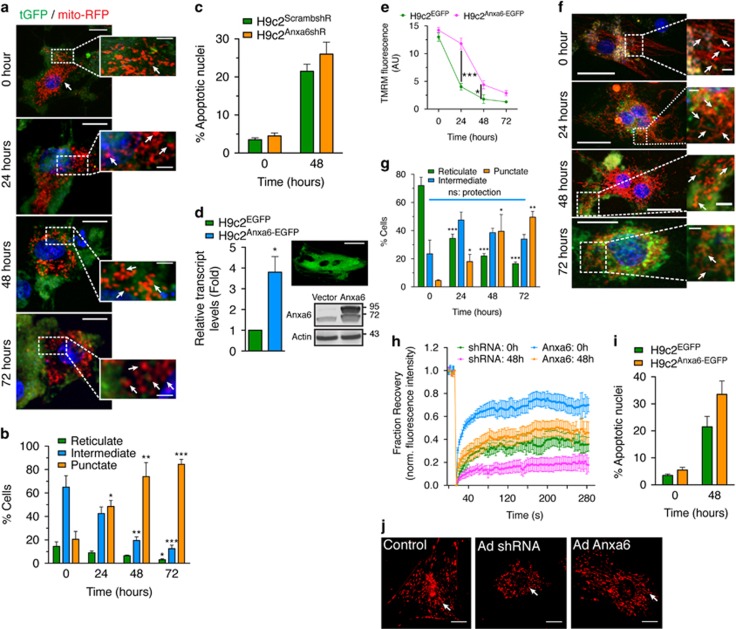
Anxa6 protects mitochondrial dynamics but increases cellular susceptibility to apoptosis. Stable cell lines with downregulated Anxa6 (H9c2^Anxa6shR^) or ectopically expressing a controlled level of Anxa6 (H9c2^Anxa6-EGFP^) were treated with PE and analyzed for the effects of Anxa6 on mitochondria and programmed cell death. (**a**) Confocal micrographs: mitochondrial morphology in H9c2^Anxa6shR^ cells expressing mito-RFP. Nuclei: Hoechst 33342. Total magnification × 600. Scale bar, 10 *μ*m. Insets, highlighted boxes. Arrowheads, mitochondrial puncta. Scale bar, 5 *μ*m. (**b**) Percentage of cells with reticulate, intermediate or punctate mitochondrial morphology in H9c2^Anxa6shR^ cells; *n*=50, ***P<0.0001; **P<0.01; *P<0.05 (with respect to *t*=0). (**c**) Percentage of H9c2^Anxa6shR^ cells with apoptotic nuclei; *n*=200 cells per group. (**d**) Fold changes of Anxa6 transcript levels in H9c2^Anxa6-EGFP^ cells; *n*=3; *P<0.05. Insets, H9c2^Anxa6-EGFP^ cell expressing Anxa6-EGFP. Magnification × 630. Scale bar, 10 *μ*m (upper panel). Immunoblot: H9c2^Anxa6-EGFP^ cells with ectopic expression of Anxa6-EGFP fusion. Actin was used as a loading control. (**e**) ΔΨm-dependent changes in TMRM fluorescence in H9c2^Anxa6-EGFP^ cells; *n*=3 experiments; ***P<0.0001; *P<0.05. (**f**) Confocal micrographs: Mitochondrial morphology in H9c2^Anxa6-EGFP^ cells. Nuclei: Hoechst 33342. Total magnification × 600. Scale bar, 10μm. Insets, highlighted boxes. Arrowheads, tubular mitochondria, Scale bar, 3 *μ*m. (**g**) Percentage of cells with reticulate, intermediate or punctate mitochondrial morphology in H9c2^Anxa6-EGFP^ cells; *n*=50; ***P<0.0001; **P<0.01; *P<0.05 (with respect to *t*=0, NS=not significant); NS, intermediate morphology representing protection. (**h**) FRAP assay for quantification of relative mobility of mitochondria in H9c2^Anxa6-EGFP^ cells infected with mito-RFP baculovirus. Plot showing normalized fluorescence intensity profiles before, during and after photobleaching (recovery fraction) for *n*=15 cells per group. (**i**) Percentage of H9c2^Anxa6-EGFP^ cells with apoptotic nuclei (*n*=3 experiments with 200 cells per group). (**j**) Fluorescence micrographs: mitochondrial morphology in NRVMs infected with control, Anxa6 shRNA or FL Anxa6 protein encoding adenovirus and cultured with PE for 48 h

Chronic PE treatment of the H9c2 cardiomyocytes progressively concentrated a sub-population of Anxa6 to mitochondria, as revealed by increased colocalization of Anxa6 (immunolabeling) with mito-RFP (Figures 5a and b). This was further supported by increased protein levels of Anxa6 in the immunoblot analysis of mitochondrial fractions at the indicated time points (Figures 5c and d). To gain mechanistic insight into the mitochondrial localization of Anxa6 in hypertrophied cardiomyocytes, the status of ΔΨm and dynamics were assessed under Anxa6-knockdown condition, using H9c2 cardiomyocytes stably expressing Anxa6 shRNA (H9c2^Anxa6shR^) or scrambled control shRNA (H9c2^ScrambshR^) (Figure 5e). Anxa6 knockdown significantly abrogated mitochondrial ΔΨm over scrambled control, as reveled by the diminished TMRM fluorescence across the period of PE treatment (Figure 5f). Furthermore, such chronic PE treatment severely affected mitochondrial motility (Supplementary Movie 4). Anxa6 knockdown cells have profuse fragmented mitochondria even in the absence of PE treatment and the percentage of fragmented mitochondria gradually increased with the duration of PE treatment (Figures 6a and b). Intriguingly, in spite of the negative effects on mitochondrial dynamics, Anxa6 knockdown seemed to have little effect on the rate of apoptosis in the hypertrophied cardiomyocytes (Figure 6c), suggesting alternative mechanisms in regulating the transition of chronic hypertrophic states towards apoptosis. However, H9c2^Anxa6shR^ cells exhibited further reduced mitochondrial connectivity after 48 h of PE treatment compared with the H9c2^ScrambshR^ cells, as measured by FRAP assay (Figure 6h).

### Ectopic expression of Anxa6 rescues mitochondrial dynamics in hypertrophied cardiomyocytes

To confirm the functions of Anxa6 in the regulation of mitochondrial dynamics and apoptosis in chronically hypertrophied cardiomyocytes, stable H9c2 cell lines ectopically expressing controlled levels of Anxa6-EGFP (H9c2^Anxa6-EGFP^) or the empty vector (H9c2^EGFP^) were generated (Figure 6d). A controlled expression level (~3- to 4.5-fold increase over control) was essential as uncontrolled overexpression of Anxa6 resulted in cell death in our pilot experiments (data not shown) and, as reported by others, leads to the development of cardiomyopathies in animal models.^19^ Analysis of TMRM fluorescence levels indicated that mitochondrial ΔΨm was significantly more conserved during PE treatment in the H9c2^Anxa6-EGFP^ cells compared with the control (H9c2^EGFP^) cells, especially during the first 24 h of treatment (Figure 6e). Live cell imaging of mitochondria showed H9c2^Anxa6-EGFP^ cells have extensive tubulature/reticulate mitochondrial network, after 24 h and significantly higher proportion of intermediate fragments after 48 h of PE treatment (Figures 6f and g). The importance of Anxa6 as an enhancer of mitochondrial dynamics was further evident in mitochondrial connectivity analysis by FRAP assay, which showed significantly higher recovery rates of mitochondrial fluorescence intensity in H9c2^Anxa6-EGFP^ cells over the control cells (Figure 6h). However, the H9c2^Anxa6-EGFP^ cells displayed higher levels of apoptotic nuclei (Figure 6i), which may be indicative of Anxa6 participating in alternate mechanisms.

To further confirm the involvement of Anxa6 in mitochondrial dynamics, hypertrophy was induced in neonatal rat ventricular cardiomyocytes (NRVMs) by PE. Hypertrophy in NRVMs was validated by immunostaining with ANP antibody showing enhanced perinuclear localization of ANP as a marker of hypertrophy after 24 h of stimulation with PE (Supplementary Figure S1G). Fragmentation of mitochondria was observed with the duration of PE treatment in NRVMs (Supplementary Figure S1H). Mitochondrial morphology was also examined in NRVMs ectopically expressing Anxa6 shRNA or full-length (FL) Anxa6. Consistent with H9c2 cardiomyocytes, mitochondrial fragmentation was also increased in hypertrophied NRVMs. Knockdown of Anxa6 further deteriorated mitochondrial morphology, which was restored almost completely when Anxa6 was overexpressed (Figure 6j).

### Anxa6 interacts with Parp1 in hypertrophied cardiomyocytes

**Figure 7:**
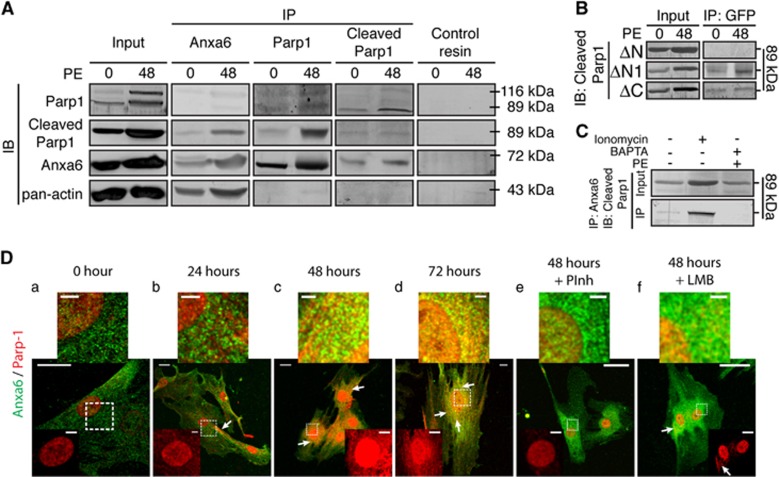
Interactions of Anxa6 with Parp1 in cardiomyocytes. H9c2 cardiomyocytes treated with PE were examined for association between Anxa6 and Parp1. (**A**) WCL from wild-type H9c2 (input), without, or treated with PE were subjected to IP, followed by immunoblot (IB) analysis of the immunoprecipitate. Antibodies for IP are indicated at the top, and for IB are indicated on the left. Pan-actin was used as an antibody control for IP and a loading control for input IB, respectively. (**B**) WCL (input) from H9c2 cells expressing Anxa6 deletion mutants indicated on left, after PE treatment, were subjected to IP, followed by IB. Living colors (JL-8) antibody was used to pull-down EGFP fusion (IP) and was probed for IB using antibody for cleaved Parp1. (**C**) WCL (input) from wild-type H9c2 (input), pretreated with ionomycin or BAPTA and without, or treated with PE for 48 h, were subjected to IP, followed by IB analysis of the Anxa6 immunoprecipitate for cleaved Parp1. (**D**) Confocal micrographs: cells double immunostained for Anxa6 (green) and Parp1 (red). Cells were treated without (a) or with PE for 24–72 h (b–d) or in combination with either PARP inhibitor (e) or Leptomycin B (f). Scale bar, 10 *μ*m. Arrowheads, cytosolic Parp1 signal and colocalized pixels. The magnified image of the white box shows perinuclear compartment with Anxa6–Parp1 distribution. Scale bar, 3 *μ*m. Inset, nuclear/perinuclear compartment showing leakage of Parp1 to cytosol. Scale bar, 2 *μ*m

Parp1 is known to have vital roles in cardiac remodeling^38^ and is activated (via cleavage) parallel to ANP secretion in response to hypertrophic stimulus.^39^ It is also instrumental in late phases of apoptosis, with Parp1 cleavage by caspases being a hallmark feature.^40^ Although Parp1 is a nuclear enzyme, the 89 kDa cleaved fragment moves to the cytosol via nucleocytoplasmic transport.^41^ Thus, we questioned whether Anxa6 interacts with Parp1 in H9c2 cardiomyocytes in course of chronic hypertrophy to bring about the differential apoptotic responses observed under ectopic Anxa6 expression. Co-immunoprecipitation (co-IP) experiments showed an increased physical association of Anxa6 with the FL as well as to a greater extent with the cleaved Parp1 under PE treatment (Figure 7A). Furthermore, N-terminal deletion mutants of Anxa6, ΔN (1–89) and ΔN1 (29–89) exhibited a differential interaction with cleaved Parp1 (Figure 7B), with ΔN showing total loss of physical interaction, whereas ΔN1 showing interaction similar to the FL. This signifies that the N-terminal residues (1–29) of Anxa6 have a crucial role in regulating its interaction with Parp1. Moreover, C-terminal mutant ΔC (600–673) showed partial binding with cleaved Parp1, presumably because of abrogated Ca^2+^ binding.^42^ Treatment of the cells with ionomycin resulted in enhanced deposition of cleaved Parp1 in Anxa6 immunoprecipitate, whereas cells pretreated with the Ca^2+^-chelator BAPTA (1, 2-Bis (2-aminophenoxy) ethane-*N*, *N*, *N*′, *N*′-tetraacetic acid tetrakis (acetoxymethyl ester)) showed complete absence of cleaved Parp1 in Anxa6 co-IP (Figure 7C). Thus, it is evident that chronic hypertrophy of H9c2 cardiomyocytes is also coupled with a Ca^2+^-dependent interaction of Anxa6 with Parp1 via its N terminus.

### Anxa6-mediated protection of mitochondrial dynamics counterbalances its interaction with Parp1

**Figure 8:**
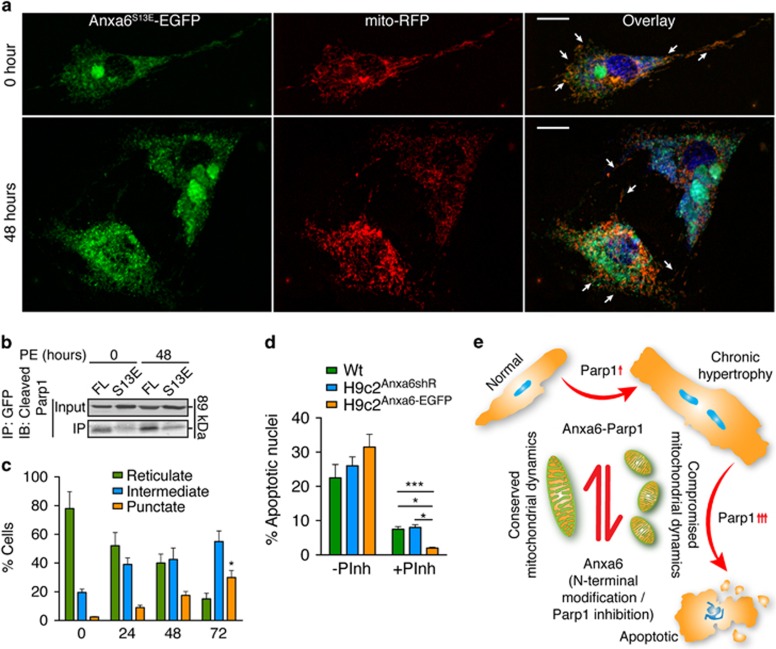
Anxa6 is essential for conservation of mitochondrial dynamics in hypertrophied cardiomyocytes. H9c2 cardiomyocytes treated with PE were analyzed for the functional involvement of Anxa6 in mitochondrial dynamics and cell death signaling. (**a**) Confocal micrographs: H9c2 cells transiently transfected with S13E mutant and infected with mito-RFP were treated with PE and analyzed for distribution of S13E with respect to mitochondria. Orange pixels in overlay channel indicate significant colocalization of Anxa6 with S13E. Arrowheads, tubular mitochondria, Scale bar, 10 *μ*m (upper panel) and 15 *μ*m (lower panel). (**b**) WCL (input) from H9c2 cells expressing Anxa6FL or Anxa6S13E, after indicated periods of PE treatment, were subjected to IP, followed by immunoblot (IB) analysis of the immunoprecipitate. (**c**) Percentage of cells with reticulate, intermediate or punctate mitochondrial morphology in H9c2^Anxa6S13E^ cells treated with PE for indicated periods; *n*=50; *P<0.05. (**d**) Percentage of cells with apoptotic nuclei after 48 h of PE treatment, without, or pretreated with PInh; *n*=3 experiments with 200 cells per group; ***P<0.0001; *P<0.05. (**e**) Schematic depiction of the dual regulatory functions of Anxa6 that mediates transition of hypertrophy to apoptosis and conserves mitochondrial dynamics in hypertrophied cardiomyocytes. Anxa6–Parp1 interaction shifts the equilibrium through chronic hypertrophy and apoptotic states and compromises mitochondrial dynamics, whereas negative regulation of Parp1 activation or modifications in Anxa6 that depletes the interaction, shifts the equilibrium away from cell death and enhances mitochondrial dynamics in hypertrophied cardiomyocytes

To analyze the functional significance of Anxa6–Parp1 interaction in hypertrophied cardiomyocytes, Parp1 localization was examined in course of PE treatment. Immunofluorescence analysis showed increased cytosolic Parp1 localization through 48–72 h. Such cytosolic localization was not observed when cells were pretreated with Parp1 inhibitor (PInh) or nuclear export inhibitor leptomycin B (LMB) (Figure 7D). It demonstrated that Parp1 activation is required for the nucleocytoplasmic transport. The co-IP results from the mutants suggested that the first 30 residues of Anxa6 seemed to be vital for the physical association with Parp1, which includes a putative phosphorylation site (S13). We found that the mutant Anxa6^S13E^ exhibited enhanced mitochondrial localization (Figure 8a) and reduced interaction with cleaved Parp1 (Figure 8b). The H9c2^Anxa6-S13E^ cells displayed enhanced mitochondrial tubulature even after 48 h of PE treatment (Figure 8c). It appears that the association of Anxa6 with Parp1 mediates activation of the later; an event that may contribute to elevated apoptotic counts in Anxa6-expressing hypertrophied cells. Treatment of the wild-type, H9c2^Anxa6-shR^ or H9c2^Anxa6-EGFP^ cells with the PInh significantly reduced the number of apoptotic nuclei (Figures 8d and e). Moreover, treatment with the inhibitor restored mitochondrial tubulature and motility in Anxa6-knockdown cells (Supplementary Movie 6), indicating a direct role of Anxa6/Parp1 in the death pathway.

## Discussion

Signaling cross-talks drive several pathophysiological conditions in mammalian cells. The spatiotemporal dynamics of signaling molecules with the subcellular compartments are crucial for efficient signal dispersal.^43, 44, 45^ The present work describes such a regulatory circuit in the chronic hypertrophy of cardiomyocytes, in which Anxa6 helps in the conservation of equilibrium between hypertrophy and apoptosis. These conclusions are based on gain- and loss-of functions approaches using H9c2 cardiomyocytes, where we characterize the phenotypes associated with hypertrophied signaling and incorporate the functions of Anxa6 in the hypertrophic process.

Here, the induction of hypertrophy by PE, Ang II or Iso for 48–72 h is described as ‘chronic' as the phenotypes at the cellular level are robust and exhibit signs of maladaptive stress response and deteriorating cell health, in comparison with treatments for 24 h. Coupled with enlarged cell size and expression of hypertrophy markers, upregulated expression of Anxa6 is observed along with its cellular relocalization (Figures 1 and 2). As described earlier,^24^ Anxa6 forms punctate aggregates in hypertrophied cardiomyocytes. Interestingly, the punctate structures are distributed in the perinuclear compartment in the later phases of stimulation (Figure 1; 48–72 h).

Anxa6 has been implicated in the regulation of mitochondrial morphology in fibroblasts.^17^ Prolonged stimulation of cardiomyocytes with the hypertrophic agonists resulted in a drop of mitochondrial ΔΨm (Figure 3a and Supplementary Figure 1). Loss or collapse of the mitochondrial ΔΨm is known to affect negatively the fused reticulate network of mitochondria, increasing the fission cycle. A dynamic balance between mitochondrial fusion and fission is crucial for the maintenance of homeostasis and a major determinant in driving the cell state towards apoptosis.^46, 47, 48^ Further examinations on whether such loss of ΔΨm causes mitochondrial punctation or loss of dynamics reveal the presence of fragmented mitochondria in PE-treated cells. Such fragmentation patterns, as also described by others,^49^ are clearly evident after PE treatment. Loss of mobility and connectivity of the organelle are evident in the live cell imaging analysis and was further quantified by FRAP assay (Figure 3 and Supplementary Figure 1 and Supplementary Movie 3). Such analysis clearly shows an increased recovery period and decreased mobile fraction in the hypertrophied cells. Thus, it is evident that chronic hypertrophy of cardiomyocytes accelerates the mitochondrial fission cycle.

The mitochondrial pathway of cell death is activated in response to the loss of mitochondrial dynamics in cardiomyocytes,^50^ and recently, the intrinsic caspase activation cascade is reported to be instrumental for the development of cardiac hypertrophy.^34^ However, whether such developments culminate to apoptotic cell death remains controversial. Furthermore, apoptotic phenotypes associated with transition of hypertrophy to heart failure^7^ does not faithfully reproduce in agonist-induced hypertrophy of isolated cardiomyocytes in culture and the available reports are contradictory.^3, 51, 52, 53, 54, 55^ This severely limits our understanding of the intricate regulatory mechanisms governing each phenomenon. Here, we characterize the programmed cell death signaling activation in course of chronic hypertrophy as a ‘mild' phenotype that elevates with longer periods of treatment. The different agonists exhibit differential behavior in their potency to induce apoptosis in our studies (Figures 4a and b), thus revealing pathway preferences in progression of hypertrophy towards apoptosis. Specifically, PE is found to be more protective in comparison with Iso or Ang II. However, activations of both ‘initiator' and ‘executioner' caspases 3 and 9, respectively, are observed in H9c2 cardiomyocytes chronically treated with PE (Figures 4c–e), which progress towards Parp1 cleavage, a hallmark of the late-phase apoptosis (Figures 4f and g). The discrepancy in activation of apoptotic machinery at the molecular levels and their execution at the cellular level could be explained by the presence of a dynamic equilibrium consisting of the counterbalancing feedback circuitry that shifts the equilibrium to a direction favorable for the maintenance of cellular homeostasis.^3^ As Anxa6 has multiple scaffolding functions, it appears to be a promising candidate for synchronizing such machineries.

Chronic PE treatment induces progressive association of Anxa6 with mitochondria (Figure 5), which is essential for preventing loss of mitochondrial dynamics in hypertrophy, as Anxa6 knockdown cells display severely abrogated mitochondrial ΔΨm and dynamics (Figures 5f and 6a, b; Supplementary Movie 4). However, ectopically expressed Anxa6 at controlled levels protects the cardiomyocytes against such loss of mitochondrial dynamics (Figures 6e and g and Supplementary Movie 5). This pattern is more evident in the FRAP analysis of mitochondrial connectivity, which displays increased mobile fraction and decreased recovery time in Anxa6-expressing cells (Figure 6h). It demonstrates that the presence of Anxa6 provides a significant level of protection against loss of mitochondrial dynamics in hypertrophied cardiomyocytes. However, subtle differences are observed in apoptotic nuclear morphology in H9c2^Anxa6-EGFP^ and H9c2^Anxa6shR^ cells (Figures 6c and i), compared with controls, with Anxa6-expressing cells having relatively higher levels of apoptotic cells at 48 h. Thus, we hypothesize that Anxa6 might be interfering with components of the dynamic equilibrium between hypertrophy and apoptosis in cardiomyocytes.

We have shown earlier that Parp1 interacts with Anxa6 in the myocardium.^23^ Here, we found that both Parp1 and its 89 kDa cleaved fragment physically bind with Anxa6 in PE-treated cardiomyocytes (Figure 7). Consistent with the earlier report, Parp1 activation in cardiomyocytes is found to be a Ca^2+^-dependent process.^39^ A progressive increase in the cytosolic signal of Parp1 was observed with increasing treatment periods, which is totally abolished by inhibition of Parp1 or nuclear export (Figure 8d), suggesting that the activation of Parp1 is instrumental for the process.

The N-terminal residues (1–29) of Anxa6 that seems crucial for interacting with Parp1 also contains a putative phosphorylation site at S13.^12^ To examine the possible roles of the posttranslational modification of Anxa6 in regulating its dynamics, the phosphomimic S13E mutant is used, which displays enhanced mitochondrial localization (Figure 8a). Binding of Parp1 with Anxa6 is abrogated in cells expressing the S13E mutant (Figure 8b). Surprisingly, the mitochondrial localization of Anxa6 is enhanced in these cells, which may be responsible for improved mitochondrial dynamics in Anxa6^S13E^ cardiomyocytes (Figure 8c). In other words, Anxa6^S13E^ technically improves the functionality of Anxa6 to act as an enhancer of mitochondrial dynamics, bypassing the Parp1-mediated shift of the equilibrium towards apoptotic state. Such counterbalancing mechanism of Anxa6 function was clearly visualized in H9c2^Anxa6shR^ cells after PE treatment and pretreatment with PInh. These cells displayed abundant mitochondrial motility and enriched intermediate fragments (Supplementary Movie 6).

To our knowledge, the present study is the first evidence where spatiotemporal dynamics of Anxa6 is shown to be critical for maintaining equilibrium between chronic hypertrophy and cell death to avoid stressful maladaptations of cardiomyocytes. However, the *in vitro* analysis of mitochondrial dynamics and cell death using experimental model of H9C2 cardiomyocytes remain a limitation of this study and whether such mechanisms operate *in vivo* warrants further investigation. In summary, we have uncovered a dual regulatory role of Anxa6, one that regulates Parp1 activation and subsequent cell death machineries and the other as an enhancer of tubular mitochondrial morphology in hypertrophied cardiomyocytes, thereby acting as a molecular switch that modulates the transition of hypertrophic phase to apoptosis. However, the former role, as described above, depends on a multitude of signaling mediators and demands further characterization. As mitochondrial dynamics is emerging as a potential new therapeutic target for heart failure,^56^ the scaffolding activity offered by Anxa6 holds much promise as a positive regulator of mitochondrial dynamics in hypertrophied cardiomyocytes.

## Materials and Methods

### Reagents

Common laboratory reagents were purchased from Life Technologies (Grand Island, NY, USA), Sigma (St. Louis, MO, USA) and Thermo Scientific (Waltham, MA, USA), unless otherwise mentioned. PE, Ang II, Iso and LMB were from Sigma. Ionomycin, BAPTA-AM, fluorescent conjugates and other microscopy consumables were from Life Technologies. Mitochondria Isolation Kit and Co-IP Kits were from Pierce Biotechnology (Rockford, IL, USA). PInh was from Calbiochem (La Jolla, CA, USA). JC-1 Staining Kit was from Cayman Chemicals (Ann Arbor, MI, USA). DAPI, Hoechst 33342, propidium podide (PI), Annexin V-Alexa Fluor 488, TMRM, MitoTracker red FM, CellLight Mitochondria-RFP and BacMam 2.0 system were from Life Technologies. Primary antibodies were procured from the following sources: Anxa6 (monoclonal antibody) from BD (Lexington, KY, USA); Anxa6 (polyclonal antibody), COX IV (cytochrome *c* oxidase subunit IV), Akt, p-AktS473 and *α*-SkA from Santa Cruz Biotechnology (Santa Cruz, CA, USA); *α*-tubulin, cleaved caspase-3, cleaved caspase-9, Parp1 and cleaved Parp1 from Cell Signaling Technology (Beverly, MA, USA); pro-ANP from Abcam (Cambridge, MA, USA); anti-COX I monoclonal antibody from Invitrogen (Carlsbad, CA, USA); Living Colors anti-fluorescent protein antibody (JL-8) from Clontech (Mountain View, CA, USA) and pan-actin from Chemicon (Temecula, CA, USA).

### Constructs

Plasmid vectors and Anxa6 shRNA constructs were acquired from Clontech and OriGene Technologies (Rockville, MD, USA), respectively. Generation of the EGFP-tagged Anxa6 constructs has been described earlier.^24^ Site-directed mutants of Anxa6 were generated using the Quik Change Lightning Kit (Stratagene, La Jolla, CA, USA) following the manufacturer's instructions. Primers used for construct generation and site-directed mutagenesis were described earlier.^24^ For generation of the mutant Anxa6S13E, the following pairs of mutagenic primers were used: (forward) 5′- GTGCCATGTACCGAGGCGAGGTCCACGACTTCGCAGA-3′ and (reverse) 5′-TCTGCGAAGTCGTGGACCTCGCCTCGGTACATGGCAC-3′. To knockdown Anxa6 in NRVMs, Anxa6 shRNA was prepared and the adenoviral vectors (kindly provided by B Vogelstein, The Johns Hopkins Medical Institutions, Baltimore, MD, USA) expressing shRNAs were generated following the guidelines for efficient siRNA designing protocol.^57^ For ectopic expression of FL Anxa6, adenoviral construct was prepared using the following primers: (forward) 5′-ACTTAAGCTTCGAGCTGCGTCTATCTGTCTGC-3′ and (reverse) 5′-CGAGTCTAGAGCACCCCAGCCGGATCACAGT-3′.

### Cell culture

H9c2 cardiomyocytes acquired from the National Centre for Cell Science (Ganeshkhind, Pune, India) were cultured in DMEM with high glucose (4.5 g/l), sodium bicarbonate (1.5 g/l) and FBS (10%) in a incubator at 37 °C, 5% CO_2_ and 80% RH. Cells were serum starved for 18–24 h before experimentation. Transfection, time frames for analysis of gene expression and generation of stable cell line have been described before.^24^ The Anxa6S13E mutant took more time in maturation and expression posttransfection. Consequently, experimentation involving Anxa6S13E was carried out 60–72 h posttransfection. Baculoviral mito-RFP transduction was carried out as per the manufacturer's protocol. Briefly, imaging assays were setup 24–28 h after the addition of virus. Hypertrophy was induced by treating serum-starved cells with 100 *μ*M PE or 300 nm Ang II or 10 *μ*M Iso for indicated time periods. For ionomycin and BAPTA treatments, cells were preincubated with either 4 *μ*M of ionomycin for a period of 10 min or with 10 *μ*M of BAPTA-AM for a period of 30 min. Pretreatment with PInh XIV was carried out with 10 *μ*M for 30 min.^58^ NRVMs from 2- to 3-day-old Sprague–Dawley rat pups were isolated as described earlier.^59, 60^

### Real-time quantitative RT-PCR

The following primers were used for quantitative real-time RT-PCR: Anxa6 (forward) 5′-GCCGCTTGCCTATTGTGAC-3′, Anxa6 (reverse) 5′-GCTGGTGTATCTGCTCATTGG-3′ 18 S rRNA (forward) 5′-CGGCTACCACATCCAAGGAA-3′, 18 S rRNA (reverse) 5′-AGCTGGAATTACCGCGGC-3′. Relative quantification of gene expression was performed as described earlier.^28, 61^ Analysis of the fold changes in transcript profiles by 2^−ΔΔCT^ method has been described elsewhere.^62^ Fold changes in the mRNA levels were normalized to 18 S rRNA levels.

### Fractionation, co-IP and western blotting

Whole-cell lysates (WCL) were made in CellLytic MT buffer (Sigma) and were supplemented with Halt protease-phosphatase inhibitor cocktail (Thermo Scientific Pierce, Waltham, MA, USA). Mitochondrial fractionation and co-IP experiments were carried out using commercially available kits (Pierce) as per the manufacturer's instructions. Briefly, 5 × 10^6^ cells were used for mitochondrial isolation and pelleted mitochondria were boiled in lysis buffer for 3 min to yield mitochondrial lysate. For co-IP experiments, 1 mg of the lysate (made with the supplied IP lysis buffer with added Halt protease and phosphatase inhibitor mixture) was subjected to IP, with 10% of the starting material (~100 *μ*g) used as an input. Antibodies were coupled onto amine-reactive resins and the lysates were incubated with these antibody-coupled resins or control resins provided with the kit overnight at 4 °C with end-to-end rotation to ensure homogeneous mixing. About 25–100 *μ*g of the elution fraction was subjected to the subsequent western blot analysis. SDS-PAGE, blotting and the detection methods with subsequent densitometric quantification were performed as described earlier.^28, 61^

### Microplate assays

All microplate assays were carried out aseptically in Special Optics black-walled 96-well plates (Corning Coster, Tewksbury, MA, USA) to reduce well-to-well cross-talks and improve the signal-to-noise ratio of data acquisition. Signal detection was carried out in a Spectramax Paradigm multimode detection platform (Molecular Devices, Sunnyvale, CA, USA) with dynamic z-stepper, operating in bottom-read mode.

### Microscopy

Confocal microscopy and live cell imaging setups and the procedure for immunocytochemistry (ICC-IF) have been described earlier.^24^ High-resolution image acquisition was performed by slow unidirectional sequential scanning (200–400 Hz; beam expander: 6) and smaller frame sizes were used for higher magnification. For time-lapse imaging, faster scanning speeds (800–1000 Hz) coupled with low line averaging (0–2) were used to preserve temporal resolution. Pixel dimensions and Z-steps were estimated to satisfy Nyquist sampling criteria. Disc spinning speed of the CSU was set to 1800 r.p.m. All comparable sets of images were acquired under identical stack parameters, laser power, detector gain, offset and pinhole aperture windows. Staining of cells with phalloidin, TMRM, JC-1, Hoechst and MitoTracker red were performed as per the manufacturer's instructions. Live cell imaging of mitochondria was performed as described elsewhere.^63^ FRAP assay was carried out using an S APO 63.0 × 1.42NA oil objective at room temperature (22 °C). One hundred percent of a 561 nm laser line was used for bleaching a small rectangular region of interest in a single scan. Recovery of fluorescence was then monitored at low laser power (1–4%) with the same laser line.

### Data processing

Image processing was performed with NIH ImageJ (NIH, Bethesda, MD, USA). Briefly, stacks of images were deconvolved and rendered into maximum intensity projections for display or average projections for quantification. For quantification purposes, images were thresholded for segmentation. Bleach correction was applied to all time stacks by using a histogram matching macro (Kota Miura and Jens Rietdorf, EMBL Heidelberg, Germany). Quantification of cellular hypertrophy in cardiomyocytes, colocalization analysis as well as particle tracking procedures has been described earlier.^24^ Analysis of mitochondrial morphology was carried out by an ImageJ macro that automates the measurement of the mitochondrial morphological parameters.^39, 40^ For FRAP analysis, background corrected images were smoothed with a median filter, and recovery in the bleached region was quantified and corrected for bleaching that occurred during acquisition at low laser power as described.^41^ The recovery (mobile fraction) was calculated as described elsewhere.^42^ All images were assembled in Adobe Illustrator (Adobe Systems, San Jose, CA, USA).

### Statistical analysis

Experiments were repeated independently at least three times and archetypal data are presented. Statistical evaluation was carried out using Prism 5 (GraphPad Software, La Jolla, CA, USA). Unless stated otherwise, data represent mean±S.E.M. Means were compared by unpaired, two-tailed Student's *t*-test between two groups or one-way analysis of variance, followed by *post hoc* comparisons with the Tukey's test between multiple groups were performed. *P*<0.05 was considered statistically significant.

## Abbreviations

Ang II: angiotensin II
ANP: atrial natriuretic peptide
Anxa6: annexin A6
α-SkA: *α*-skeletal actin
BAPTA-AM: 1, 2-Bis (2-aminophenoxy) ethane-*N*, *N*, *N*′, *N*′-tetraacetic acid tetrakis (acetoxymethyl ester)
Ca^2+^: calcium
Co-IP: co-immunoprecipitation
COX I: anti-OxPhos complex IV subunit I
COX IV: cytochrome *c* oxidase subunit IV
ΔΨm: mitochondrial membrane potential
FL: full length
FRAP: fluorescence recovery after photobleaching
IB: immunoblot
IP: immunoprecipitation
Iso: isoproterenol
LMB: leptomycin B
NRVM: neonatal rat ventricular myocyte
Parp1: poly-(ADP-ribose) polymerase 1
PE: phenylephrine
PI: propidium iodide
PInh: Parp1 inhibitor
pro-ANP: propeptide of atrial natriuretic peptide/intracellular precursor of atrial natriuretic peptide
Scramb: scrambled control
TMRM: tetramethylrhodamine
WCL: whole-cell lysate
