# Supplementary material for: Balancing functions of annexin A6 maintain equilibrium between hypertrophy and apoptosis in cardiomyocytes
Source: Cell Death Dis. 2015 Sep 3;6(9):e1873–. doi: 10.1038/cddis.2015.231 (PMC4650436; doi:10.1038/cddis.2015.231)
Supplement: Supplementary Figure and Movie Legends [file cddis2015231x1.pdf]

SUPPLEMENTARY FIGURE AND MOVIE LEGENDS

FIGURE S1. A, Quantitative analysis of Δψm dependent changes in TMRM fluorescence at indicated time periods after treatment with hypertrophic agonist. STS was used as positive control. Data represent mean ± S.E.M of n=3 experiments; *** and ### indicate $p < 0.0001$; **, $p < 0.\ 01$; * and #, $p < 0.05$ (*, t=0; #, t=24; ns = not significant). **B,** Plot showing ratio of red to green channels from JC-1 stained cells shown in B; n=30 fields; ***, $p < 0.0001$; #, $p < 0.05$ (*, t=0; #, t=24). **C,** Confocal micrographs showing cells treated for indicated periods and immunostained for COX I to identify mitochondrial morphology. Nuclei were counterstained with DAPI. Scale bar, 10 µm. Insets show high-resolution images of dotted boxes. Scale bar, 1 µm. Arrowheads in left and right panels indicate reticulate and punctate mitochondrial morphology. **D,** Live cell imaging for categorization of different mitochondrial morphology observed in hypertrophied cardiomyocytes treated with hypertrophic agonists and stained with MitoTracker red. Scale bar, 10 µm. Insets show magnified view of dotted boxes. Scale bar, 5 µm. **E,** Total area occupied by mitochondria per cell versus mitochondrial area as percentage of total cellular area. **F,** Micrographs showing nuclear morphology in cells stained with Hoechst 33342 after treatment with indicated agonists and time periods. Note appearance of nuclear condensation beyond 48 hours. PE treated cells showed significantly lower percent of apoptotic nuclei than cells treated with Ang II or Iso. 630X total magnification, Scale bar, 30 µm. Arrowheads, condensed nuclei. **G,** Confocal micrographs showing NRVM without (control) or treated with PE and immunostained for ANP (green). Nuclei counterstained with DAPI (blue). 630X total magnification, scale bar, 15 µm. Arrowheads indicate perinuclear ring of ANP. **H,** Fluorescence micrographs showing NRVM stained for MitoTracker Red after indicated hours of PE treatment. 400X maginification. Arrowheads indicate tubular or fragmented mitochondria, scale bar, 20 µm.

Movie 1. Wild type H9c2 cardiomyocytes at 0 hours of PE treatment. 3D rendered AFM images of live H9c2 cardiomyocytes. Cells were serum starved for 18 hours. Scale bar, Pseudocolored LUT bar (inset). Movie compiled at 7 frames per second (fps).

Movie 2. Wild type H9c2 cardiomyocytes after 48 hours of PE treatment. 3D rendered AFM images of live serum-starved H9c2 cardiomyocytes after treatment with PE for 48 hours. Scale bar, Pseudocolored LUT bar (inset). Larger frame size accommodated for augmented cellular volume. Movie compiled at 18 fps.

Movie 3. Morphological dynamics of mitochondria in chronically treated hypertrophied cardiomyocytes. Time lapse live cell confocal imaging of mitochondrial dynamics in H9c2 cardiomyocytes loaded with MitoTracker Red, after treatment with PE for A: 0 hour, B: 24 hours, C: 48 hours and D: 72 hours. Note regressive mitochondrial tubulature, increased punctation and loss of motility across PE treatment. Scale bar, 5 µm. Movie compiled at 6 fps.

Movie 4. Mitochondrial dynamics in hypertrophied cardiomyocytes expressing Anxa6 shRNA. Time lapse live cell confocal imaging of mitochondrial dynamics in cardiomyocytes transfected with Anxa6 shRNA (pseudocolored green) and infected with mito-RFP (pseudocolored red) baculovirus, after treatment with PE for 48 hours. Expression of tGFP (pseudocolored green) was monitored as marker of shRNA expression. Note severely abrogated mitochondrial dynamics, increased punctation and loss of motility in the cell with tGFP expression (inset). The neighboring cell with tGFP signal below the threshold visibly has better mitochondrial motility (right of magnified section) and serves as an internal control for addressing paracrine-signaling artifacts qualitatively. Scale bar, 10 µm. Movie compiled at 51 fps.

Movie 5. Morphological dynamics of mitochondria in hypertrophied H9c2Anxa6-EGFP cardiomyocytes.

Time lapse live cell confocal imaging of mitochondrial dynamics in H9c2Anxa6-EGFP cardiomyocytes infected with mito-RFP (pseudocolored red) baculovirus, after treatment with PE for 48 hours. EGFP (pseudocolored green) was monitored as marker of Anxa6 expression. Note conserved mitochondrial dynamics, tubulature and motility (inset), even after 48 hours of PE treatment. Scale bar, 10 µm. Movie compiled at 5 fps.

Movie 6. Parp1 inhibition conserves mitochondrial dynamics in chronic hypertrophic induction of H9c2Anxa6shR cardiomyocytes. H9c2Anxa6shR cells pretreated with Parp1 inhibitor and treated with PE for 48 hours have fragmented mitochondria but partially conserved morphological dynamics, compared with untreated H9c2Anxa6shR cells (supplemental Movie 7). Scale bar, 20 µm. Insets, Magnified views of regions marked. Scale bars, 5 µm (right panel); 1 µm (left panel). Movie compiled at 6 fps.
