# Supplementary figures and images for: Balancing functions of annexin A6 maintain equilibrium between hypertrophy and apoptosis in cardiomyocytes
Source: Cell Death Dis. 2015 Sep 3;6(9):e1873–. doi: 10.1038/cddis.2015.231 (PMC4650436; doi:10.1038/cddis.2015.231)

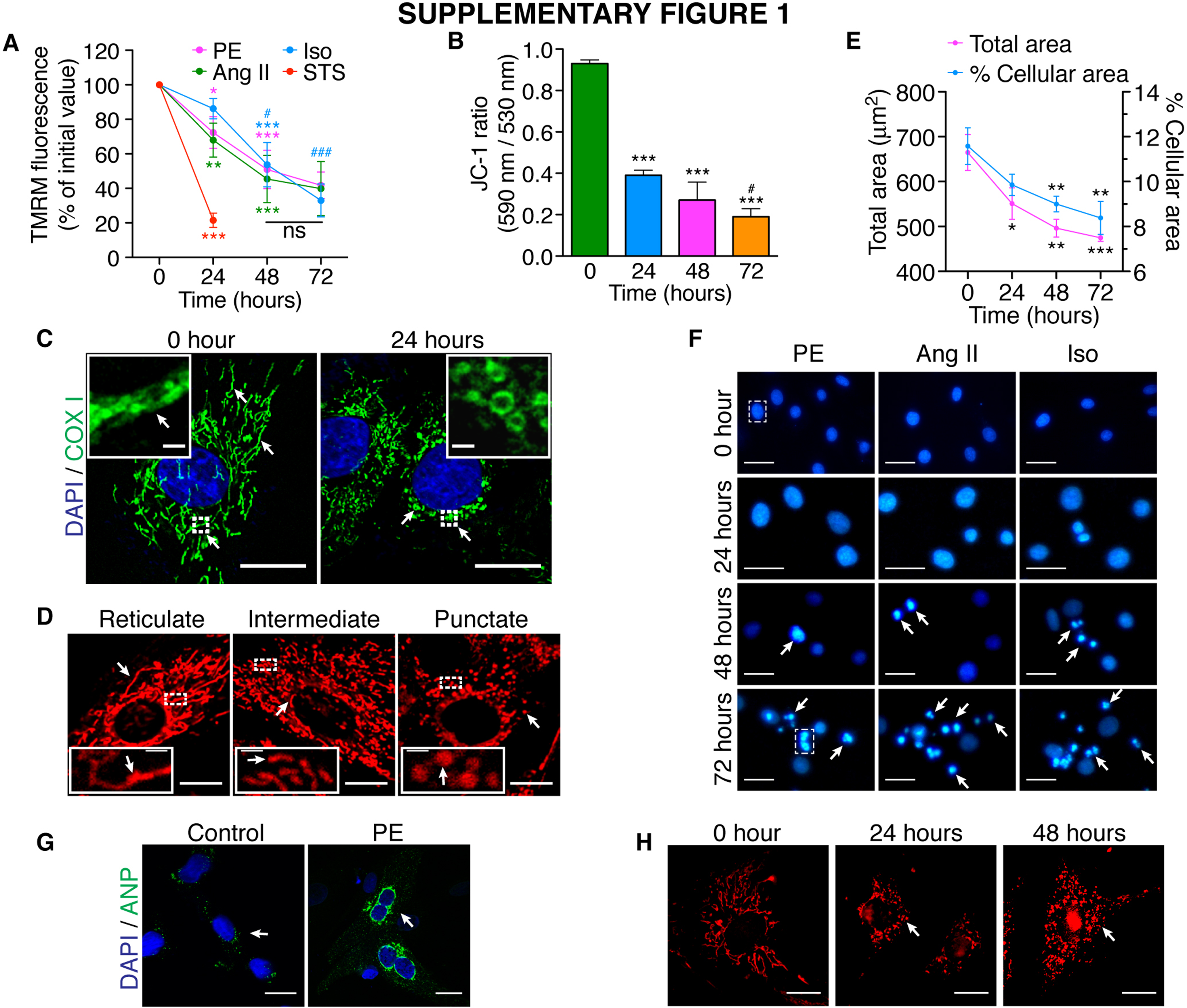

Supplement: Supplementary Figure S1 [file cddis2015231x2.tif]
